# Arginine-vasopressin deficiency due to long COVID-associated infundibulo-neurohypophysitis

**DOI:** 10.20945/2359-4292-2024-0168

**Published:** 2025-03-24

**Authors:** Regina S. Medeiros, Lígia Neves, Isabel Sousa, Bernardo Dias Pereira

**Affiliations:** 1 Serviço de Endocrinologia e Nutrição, Hospital do Divino Espírito Santo, Ponta Delgada, Açores, Portugal; 2 Serviço de Neurorradiologia, Hospital Garcia de Orta, Unidade Local de Saúde de Almada-Seixal, Almada, Setúbal, Portugal

## Abstract

Long COVID is defined by the occurrence of signs, symptoms, and conditions that develop after COVID-19 and may affect several organs and systems. Arginine-vasopressin deficiency (AVP-D; central diabetes insipidus) is a very rare complication of COVID-19 and SARS-CoV-2 immunization. Case reports, original studies, and reviews on AVP-D and long COVID published until February 2024 were retrieved from PubMed. A 47-year-old man presented with polydipsia, polyuria, memory loss, and mental fog 8 weeks after an episode of mild COVID-19. His past personal and family medical history were unremarkable. Biochemical evaluation was relevant for low urine osmolality and a 24-hour urine volume of 10,350 mL. Basal anterior pituitary evaluation was normal. A water deprivation test was started and interrupted after 2 hours due to the development of hypernatremia, high serum osmolality, and low urine osmolality. Urine osmolality significantly increased after intranasal desmopressin 20 µg. Contrast-enhanced pituitary MRI was suggestive of infundibulo-neurohypophysitis. Further biochemical, genetic, and imaging tests excluded secondary AVP-D causes.The patient was subsequently started on oral desmopressin, showing prompt response. After a follow-up of 20 months, he remained well-controlled with isolated AVP-D. Although molecular and histologic confirmation of SARS-CoV-2 infundibulo-neurohypophysitis could not be investigated, a strong temporal relationship and the absence of an alternative diagnosis rendered plausible the inclusion of AVP-D in the myriad of long COVID manifestations. Further studies with patients recovered from COVID-19 are necessary for a better understanding of the epidemiology, pathophysiology, and clinical course of this very rare endocrine condition.

## INTRODUCTION

Severe acute respiratory syndrome Coronavirus 2 (SARS-CoV-2) causes Coronavirus disease 2019 (COVID-19), a highly contagious infection that primarily leads to pulmonary complications but also triggers a myriad of extrapulmonary disturbances, affecting the gastrointestinal, hepatobiliary, cardiovascular, renal, and central nervous systems (^1^). Long COVID is defined by the World Health Organization (WHO) as a medical condition that usually occurs after 3 months of confirmed or suspected COVID-19, lasts at least 2 months, can be of new onset or persistent after initial illness, and cannot be attributed to any other condition. Frequent manifestations of long COVID include fatigue, shortness of breath, and cognitive dysfunction, but other signs and symptoms suggestive of dysfunction in several other organs may also be present (^2^). The SARS-CoV-2 uses the angiotensin-converting enzyme 2 receptor (ACE2R) to enter and infect cells, and several nonendocrine and endocrine organs express ACE2R, including the hypothalamus (median eminence capillaries and paraventricular nucleus) and the pituitary (^3^). Autopsy studies have previously shown the SARS-CoV genetic sequence in degenerated hy-pothalamic neurons (^4^). Additionally, SARS-CoV-2 genome has been found in the cerebrospinal fluid (^5^). Two possible mechanisms could explain hypothalamic-pituitary dysfunction resulting from SARS-CoV-2 infection: inflammation-mediated or direct viral hypothalamic damage (^3^).

Arginine-vasopressin (AVP) deficiency (AVP-D) is a very rare complication of COVID-19 (^6,7,8,9,10,11,12,13,14,15^). Considering the WHO criteria for long COVID-associated disturbances, only two AVP-D cases have been previously reported (^6,14^). We report herein an additional case of AVP-D associated with long COVID and briefly review the literature on this association.

## METHODS

The water deprivation test protocol was performed and interpreted as previously published (^16,17^). Magnetic resonance imaging (MRI) was performed using MAGNETOM Skyra 3T MRI (Siemens Healthineers, Erlangen, Germany) with contrast gadolinium and 2 mm slice thickness, and reference measures for adult pituitary stalk were considered as previously published (^18^). All the investigations and therapies were performed after informed consent was obtained from the patient.

We performed a computer-assisted search to identify case reports, original studies, and reviews in the English literature on AVP-D and long COVID, published in PubMed until February 2024. We retrieved data using the following keywords: diabetes insipidus, neurogenic; post-acute COVID-19 syndrome; long COVID; hypophysitis, pituitary gland, posterior; pituitary gland.

## RESULTS

### Case report

A 47-year-old man was diagnosed in January 2022 with a mild COVID-19 infection confirmed by reverse transcription polymerase reaction (RT-PCR). He reported back pain and myalgia that subsided over a period of 5 days with home treatment. Except for untreated dyslipidemia, he had no remarkable personal or family medical history. Eight weeks after the COVID-19 diagnosis, the patient was referred to the endocrinology department due to polydipsia, polyuria, nocturia, and weight loss of 6 kg. He also reported memory loss and mental fog. His physical examination was unremarkable. Biochemical analysis showed normal general parameters, except for a urine osmolality of 160 mOs/kg H_2_O and a 24-hour urine volume of 10,350 mL (Table 1).

**Table 1:** Biochemical test results of the patient, including screening for secondary causes of arginine-vasopressin deficiency (AVP-D)†

Parameter	Result	Reference
Hemoglobin (g/dL)	14.4	14-18
White blood cell count (x10^3^/µL)	6.4	4.0-11.5
Platelets (x10^3^/µL)	271	150-400
ESR (mm)	12	0-15
Fasting blood glucose (mg/dL)	98	74-106
HbA1c (%)	5.7	<6.5
Serum sodium (mmol/L)	143	135-145
Serum potassium (mmol/L)	3.8	3.5-5.1
Serum calcium (mg/dL)	9.6	8.3-10.6
Serum creatinine (mg/dL)	0.88	0.67-1.17
AST (U/L)	30	<34
ALT (U/L)	32	10-49
GGT (U/L)	18	<73
Serum copper (µg/L)	113.6	70-140
Serum iron (µg/dL)	204	65-175
Ferritin (ng/mL)	449.6	22-322
Transferrin saturation (%)	68	15-45
Serum cortisol (8 am) (µg/dL)	12.8	3.7-19.4
Stimulated serum cortisol (µg/dL)^‡^	26	(<18)
IGF-1 (ng/mL)	182.7	81-282
FSH (mIU/mL)	3.0	1.5-12.4
LH (mIU/mL)	2.53	1.7-8.6
Total testosterone (ng/dL)	394.7	249-836
Prolactin (ng/mL)	13.4	4.0-15.2
TSH (µIU/mL)	2.33	0.35-4.94
Free T4 (ng/dL)	0.95	0.7-1.48
Beta hCG (U/L)	<2.00	<3
AFP (ng/mL)	2.05	0.89-8.78
ACE (U/L)	11.8	8.3-21.4
IgG4 (mg/dL)	114	3-201
IGRA^±^	Negative	Negative
Serum osmolality (mOsm/kg)	285	275-295
24-hour urine osmolality (mOsm/kg)	160	500-850
Total urine volume (L/24h)	10.35	

Abbreviations: ACE, angiotensin-converting enzyme; AFP, alpha-fetoprotein; ALT, alanine aminotransferase; AST, aspartate aminotransferase; AVP-D, arginine-vasopressin deficiency; beta hCG, human chorionic gonadotropin beta subunit; ESR, erythrocyte sedimentation rate; FSH, follicle-stimulating hormone; GGT, gamma-glutamyl transferase; HbA1c, hemoglobin A1c; IGF-1, insulin-like growth factor type 1; IGRA, interferon-gamma release assay; LH, luteinizing hormone; T4, thyroxin; TSH, thyroid-stimulating hormone.

^†^ Secondary causes of AVP-D screened: sarcoidosis, tuberculosis, IgG4 hypophysitis, Wilson’s disease, hemochromatosis, and germinoma.

^‡^ Serum cortisol value at 60 minutes post-Synacthen (250 mg/mL; rapid test).

^±^ ELISPOT (TSPOT-TB) technique *(Mycobacterium tuberculosis* ESAT-6 and CFP10 antigens).

**Figure 1:**
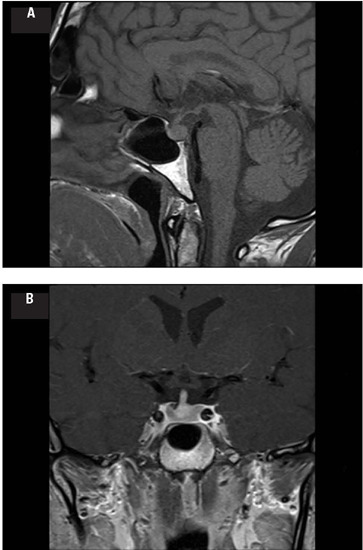
Pituitary magnetic resonance imaging (MRI) T1-weighted images showing **(A)** an absence of the posterior pituitary bright spot on sagittal section and **(B)** pituitary stalk thickening at the pituitary insertion (3.95 mm; reference [mean ± standard deviation]: 1.91 ± 0.4 mm) (^17^) on coronal section.

The patient was admitted to the endocrinology ward to undergo a water deprivation test. The test started at 8 am (water deprivation phase) but was interrupted after 2 hours due to the development of hypernatremia reflected by a serum sodium level of 146 mEq/L, along with serum osmolality of 311 mOs/kg H_2_O and urine osmolality of 105 mOsm/kg H_2_O. After 20 µg of intranasal desmopressin, his urine osmolality increased over 4 hours to 357 mOsm/kg H_2_O. Gadolinium-enhanced MRI revealed an absence of the posterior pituitary bright spot on T1-weighted images associated with a thickening of the pituitary stalk (Figures 1A and 1B), suggestive of infundibulo-neurohypophysitis.

The diagnosis of AVP-D was considered, and the patient was started on 0.2 mg of oral desmopressin at bedtime. After showing a prompt clinical response with the disappearance of nocturia, the dose was titrated to 0.8 mg/day and administered in three divided doses for improved diurnal symptoms. Screening for secondary causes of AVP-D was negative, except for elevated serum iron and transferrin saturation (Table 1). Genetic screening of hemochromatosis by next-generation sequencing of all hemochromatosis-associated genes (HFE, *HJV, HAMP, TFR2,* and *SLC40A1)* revealed a heterozygotic variant (rs1799945) c.187C>G p.(His63Asp) in HFE, a genotype not associated with iron overload. Radiographs of the chest, jaw, and cranium (to exclude Langerhans cell histiocytosis) were also normal.

In his last appointment in November 2023, the patient exhibited no symptoms of AVP-D on the same dose of desmopressin but due to complaints of persistent memory loss and mental fog, the diagnosis of long COVID was presumed. Biochemical results revealed normal sodium (140 mEq/L) and urine osmolality (415.4 mOsm/kg H_2_O), and slightly elevated serum osmolality (302.9 mOsm/kg H_2_O). Reevaluation of basal anterior pituitary function was normal.

## DISCUSSION

We presented herein the case of a male patient with long COVID-associated AVP-D. Although the molecular and histologic diagnosis of SARS-CoV-2 infundibulo-neurohypophysitis could not be confirmed, a strong temporal relationship and the absence of an alternative diagnosis rendered plausible the inclusion of AVP-D in the myriad of manifestations of long COVID.

Ten cases of AVP-D associated with COVID-19 have been previously reported (^6,7,8,9,10,11,12,13,14,15^). The main demographic, clinical, imaging, and treatment aspects of these patients are outlined in Table 2. Most cases (7 of 9 patients, 78%) were diagnosed with moderate or severe forms of COVID-19 (^6,8,9,10,11,13,14,15^), although conclusions regarding a correlation between the severity of COVID-19 and the development of AVP-D cannot be drawn due to the rarity and small number of published cases of AVP-D occurring after COVID-19. The natural history of this form of AVP-D is also difficult to determine, as most cases (7 of 9 reports, 78%) lacked follow-up or were followed (3 of 9 patients, 33%) for short periods (median 4.5 months, range 1-24 months) after the diagnosis of AVP-D (^6,7,8,14^). Thus, in addition to our case, only two other published reports (^6,14^) have complied with the WHO criteria of AVP-D as part of the spectrum of long COVID. In our case, the long-term follow-up (20 months) and the structural MRI abnormal findings in the posterior pituitary are strong evidence of the permanent nature of AVP-D, and we can likely conclude the same in cases where a loss of the posterior bright spot on T1-weighted MRI imaging was documented (^6,12^). Considering the above-mentioned follow-up limitations of the reported cases, only two patients (22%) with COVID-19-associated AVP-D showed reversibility of this pituitary dysfunction - one at 5 days and the other at 30 days after the AVP-D diagnosis (^13,14^).

**Table 2:** Demographic, clinical, imaging, and treatment aspects of published cases (including the present case) of arginine-vasopressin deficiency (AVP-D) associated with COVID-19

Reference	Age	Sex	Time between COVID-19 and endocrine symptoms (weeks)	Pituitary MRI findings	Pituitary function findings	Treatment
Present case	47	M	8	Loss of posterior bright spot Thickened stalk	AVP-D	Desmopressin
(^6^)	60	F	8	Loss of posterior bright spot Thickened stalk	AVP-D	Desmopressin
(^7^)	28	M	5	Normal	AVP-D	Desmopressin
(^8^)	54	F	6	Normal	AVP-D	Desmopressin
(^9^)	39	F	4	NA	AVP-D and resistance	Desmopressin Hydrochlorothiazide Indomethacin
(^10^)	68	M	3	Normal	AVP-D	Desmopressin
(^11^)	44	F	3	Normal	AVP-D Hypocortisolism	Desmopressin Hydrocortisone
(^12^)	17	M	3	Loss of posterior bright spot Thickened stalk	AVP-D	Desmopressin
(^13^)	35	M	2	NA	AVP-D	Desmopressin
(^14^)	16	F	3	Pituitary enlargement	AVP-D Hypocortisolism	Methylprednisolone
(^15^)	32	M	10	Loss of posterior bright spot	AVP-D	Desmopressin Methylprednisolone

Abbreviations: AVP-D, arginine-vasopressin deficiency; F, female; M, male; MRI, magnetic resonance imaging; NA, not available.

Interestingly, a transient form of mixed AVP-D/ resistance can occur in patients with COVID-19 in intensive care units (ICUs). The main causes of this transient dysfunction seem to be AVP resistance due to downregulation of V2 receptors in the kidney associated with the use of supraphysiologic intravenous vasopressin applied for several indications in ICU patients, but also AVP-D due to COVID-19-associated posterior pituitary direct or indirect (delayed immune response) injury, endotoxin-mediated depletion of vasopressin stores in septic shock, and impaired baroreceptor-mediated vasopressin secretion (^19^). Additionally, this mixed form of AVP-D/resistance may explain the polyuria that occurred in one of the cases included in our literature review (^9^).

Abnormalities of anterior pituitary function associated with COVID-19 have been reported mainly in patients with pituitary apoplexy after the diagnosis of SARS-CoV-2 infection, and AVP-D is usually not present in these cases (^20^). Establishing a relationship between COVID-19 and anterior pituitary dysfunction is challenging, as many patients reported with this possible association have been submitted to prolonged supraphysiologic doses of glucocorticoids for COVID-19 treatment (^21^). However, there are very few cases in which the relationship between anterior pituitary dysfunction and COVID-19 was unaffected by the bias introduced by glucocorticoid use, including cases of isolated secondary adrenal insufficiency (^22^) or combined deficiency of ACTH, gonadotropins, and growth hormone (^23^). In our literature review, only two patients with COVID-19-associated AVP-D had secondary adrenal insufficiency, and one of them recovered from both anterior and posterior pituitary deficiencies after a course of highdose methylprednisolone (^11,14^).

Posterior pituitary dysfunction related to central nervous system (CNS) infections other than COVID-19 is also rare and limited to case reports (^24^). The largest published cohorts of patients with CNS infections unrelated to COVID-19 (*e.g.,* due to viral, fungal, or bacterial etiologies) and causing pituitary involvement have not reported the occurrence of AVP-D. However, anterior pituitary deficiencies are seen in as much as 31% of cases, primarily involving GH deficiency, hypocortisolism, and hypogonadism (^24^), and they may be evident only 12 months after the acute infection, justifying additional follow-up and hormonal testing (^24^).

Notably, AVP-D has also been reported after SARS-CoV-2 immunization. Most of such cases (Table 3) affected female patients (6 of 8 patients) a few days after the inoculation, and the use of the BNT162b2 vaccine was reported in 6 of 8 patients (^25,26,27,28,29^). Most cases (6 of 8 patients) presented with isolated AVP-D and two patients (25%) had hypopituitarism (^30^). The MRI findings of thickened pituitary stalk and/or loss of posterior bright spot were seen in almost all cases (7 of 8 patients). There are several proposed pathophysiologic mechanisms for AVP-D developing after SARS-CoV-2 immunization, of which the most frequent is autoimmune/inflammatory syndrome induced by adjuvants of vaccines, with consequent molecular mimicry and cross-reaction with natural antigens (^29,30^).

**Table 3:** Demographic, clinical, imaging, and type of vaccine in published cases of arginine-vasopressin deficiency (AVP-D) associated with SARS-CoV-2 immunization

Reference	Age	Sex	Time between SARS-CoV-2 immunization and endocrine symptoms (days)	Pituitary MRI findings	Pituitary function findings	Vaccine
(^27^)	51	M	3	Pituitary enlargement	AVP-D Hypocortisolism Hypothyroidism Hypogonadism	BNT162b2
(^25^)	59	F	56	Loss of posterior bright spot Thickened stalk	AVP-D	BNT162b2
(^27^)	37	F	7	Loss of posterior bright spot	AVP-D	BNT162b2
(^27^)	48	F	2	Thickened stalk Empty sella	AVP-D GH deficiency Hypothyroidism Hypogonadism	BNT162b2
(^28^)	16	M	NA	Loss of posterior bright spot Thickened stalk	AVP-D	BNT162b2
(^27^)	54	F	3	Thickened stalk	AVP-D	ChAdOx1
(^26^)	74	F	30	Loss of posterior bright spot Thickened stalk Pituitary enlargement	AVP-D	Spikevax
(^29^)	21	F	7	Loss of posterior bright spot Thickened stalk	AVP-D	BNT162b2

Abbreviations: AVP-D, arginine-vasopressin deficiency; F, female; GH, growth hormone; MRI, magnetic resonance imaging; M, male; NA, not available.

In conclusion, our case further strengthens the inclusion of AVP-D in the myriad of manifestations of COVID-19 and long COVID. Further studies of patients recovered from COVID-19 who present with AVP-D, ideally including histologic data and longer follow-up periods, are necessary for a better understanding of the pathophysiology and clinical course of this endocrine complication of COVID-19 and long COVID.
